# An efficient method for noninvasive prenatal diagnosis of fetal trisomy 13, trisomy 18, and trisomy 21

**DOI:** 10.1371/journal.pone.0215368

**Published:** 2019-04-12

**Authors:** Xiaohan Sun, Jianbo Lu, Xu Ma

**Affiliations:** 1 Tianjin Polytechnic University, Tianjin, China; 2 Human Genetics Resource Center, National Research Institute for Family Planning, Beijing, China; 3 Graduate School, Peking Union Medical College, Beijing, China; Consiglio Nazionale delle Ricerche, ITALY

## Abstract

**Background:**

Molecular size determination of circulating free fetal DNA in maternal plasma is an important detection method for noninvasive prenatal testing (NIPT). The fetal DNA molecule is the primary factor determining the overall performance of NIPT and its clinical interpretation. The proportion of cell-free fetal DNA molecules is expressed as the fetal DNA fraction in the plasma of pregnant women.

**Methods:**

We proposed an effective method to deduce fetal chromosomal aneuploidy based on the proportion of a certain range of DNA fragment lengths from maternal plasma. We gradually narrowed the range of the upper and lower boundary via a traversing algorithm.

**Results:**

We explored the optimal range of the upper and lower boundary by using size-based DNA fragment length. Using this range, the accuracy of the sensitivity and specificity could be improved by up to 100% for detecting the three most common autosomal aneuploidies, namely trisomy 13, trisomy 18, trisomy 21 in the sample set.

**Conclusions:**

Numerical experiments demonstrate that our method is effective and efficient. The program is available upon request.

## Introduction

Noninvasive prenatal testing (NIPT) is now widely used in clinical practices worldwide and does not require traumatic sampling, with high accuracy, high sensitivity and specificity. For most pregnant women, the invasive sampling of fetal genetic material through amniocentesis or chorionic villus sampling is gradually replaced by noninvasive prenatal testing [1,2]. NIPT can screen for fetal chromosome aneuploidy and certain copy number variations [2]. In 1997, Dennis Lo found that cell-free fetal DNA (cffDNA) was present in the maternal plasma and it increased stably with the gestational weeks and disappeared rapidly with the delivery of pregnant women, which can be used as an ideal material for NIPT [3]. The cffDNA was discovered in plasma of pregnant women in 1997 which developed a new technique to analyze and measure accurately fetal DNA in maternal plasma [3].

Detection of the three most common autosomal aneuploidies is an important indication for noninvasive prenatal diagnosis [4]. The three aneuploidies are trisomy 21 syndrome (Down syndrome), trisomy 13 syndrome (Patau syndrome) and trisomy 18 syndrome (Edwards syndrome) [5]. In recent years, high accuracy has been achieved in prenatal screening for Down syndrome. However, identification of trisomy 13 and trisomy 18 remains a significant secondary goal [6]. Down syndrome occurs in 1 in 800 live births, whereas about 1 in 10,000 newborns is estimated to carry trisomy 13, and the incidence of trisomy 18 is about 1 out of every 6,000 live births [6,7]. Due to Down syndrome and Patau syndrome, as well as Edwards syndrome with high rates of spontaneous loss, live-born infants are less likely to survive beyond the early stage [8]. Only 5–10% of live-born infants with the three aneuploidies can survive more than one year [9,10].

A new generation of DNA sequencing technology is used to sequence the cell-free DNA fragments in the maternal plasma. The sequencing results are subjected to bioinformatics diagnostic algorithms for obtaining the fetal genetic information and detecting the presence of the three most common autosomal aneuploidies [11–15]. With the availability of massively parallel sequencing technologies, some applied data can be obtained, such as the genomic identities and quantities of millions of DNA molecules in biological samples originating from the plasma of pregnant women [4]. To date, a series of new avenues of noninvasive diagnostic applications have developed; for example, chromosomal aneuploidy detection, fetal sex determination, and detection of monogenic diseases [16–18]. The cffDNA contained in the maternal plasma accounts for about 5%-20% of the total cell-free DNA fragments [19–21]. The fractional fetal DNA concentration is a paramount factor for determining the overall performance of NIPT based on the analysis of DNA in maternal plasma.

In previous studies, the detection of fetal chromosomal aneuploidy has been discussed, but its processing is cumbersome and complicated. Most studies take advantage of the fetal DNA fraction to analyze sequencing data of maternal plasma DNA though various methods and instruments. In this study, we proposed a simple and effective method, which employs a range of cell-free DNA segment lengths to determine fetal chromosomal aneuploidy. A chromosomal abnormality would lead to an increased or reduced representation of the chromosome in the contents of cell-free DNA fragments in the maternal plasma [22,23]. If a woman is pregnant with a trisomic fetus, the amounts of its corresponding chromosome fragments should be increased [4,22]. Because of the extra chromosome, the proportion of corresponding cell-free DNA fragments would also be elevated in the maternal plasma when compared to a pregnancy with a euploid fetus. Based on this, the study discussed the suitable range of cell-free DNA fragment lengths derived from maternal plasma for the detection of fetal chromosomal aneuploidy.

## Materials

One sample set was applied which had been used in previous studies [2,4–5]. There were 144 maternal plasma sample cases, which were divided into four parts in the sample set. These included 21 cases each with a trisomy 13 fetus, 27 cases each with a trisomy 18 fetus, 36 cases each with a trisomy 21 fetus, and 60 cases each with a euploid fetus. None of the pregnancy samples collected were tested for any invasive sampling of fetal genetic material [5]. The sample set we used came from “Size-based molecular diagnostics using plasma DNA for noninvasive prenatal testing”, contributed by Y. M. Dennis Lo, April 2, 2014, PNAS. USA. The data information is shown in S1 Dataset.

## Methods and results

### Calculating the proportion of chromosomes

First, we calculated the percentage of each chromosome contained in the sample set separately. The sample set contained the size distribution of cell-free DNA fragments originating from maternal plasma. The following formula was used to calculate the proportion of every corresponding chromosome, and the result was shown in S1 Appendix.

$$P_{chrN}=\frac{Sum_{chrN}}{Sum_{all}}$$

Where *P*_*chrN*_ denotes the proportion of target chromosomes fragments in all chromosomes fragments originating from maternal plasma samples; *Sum*_*chrN*_ denotes the sum of target chromosomes fragments originating from maternal plasma samples; and *Sum*_*all*_ denotes the sum of all chromosomes fragments originating from maternal plasma samples.

**Fig 1:**
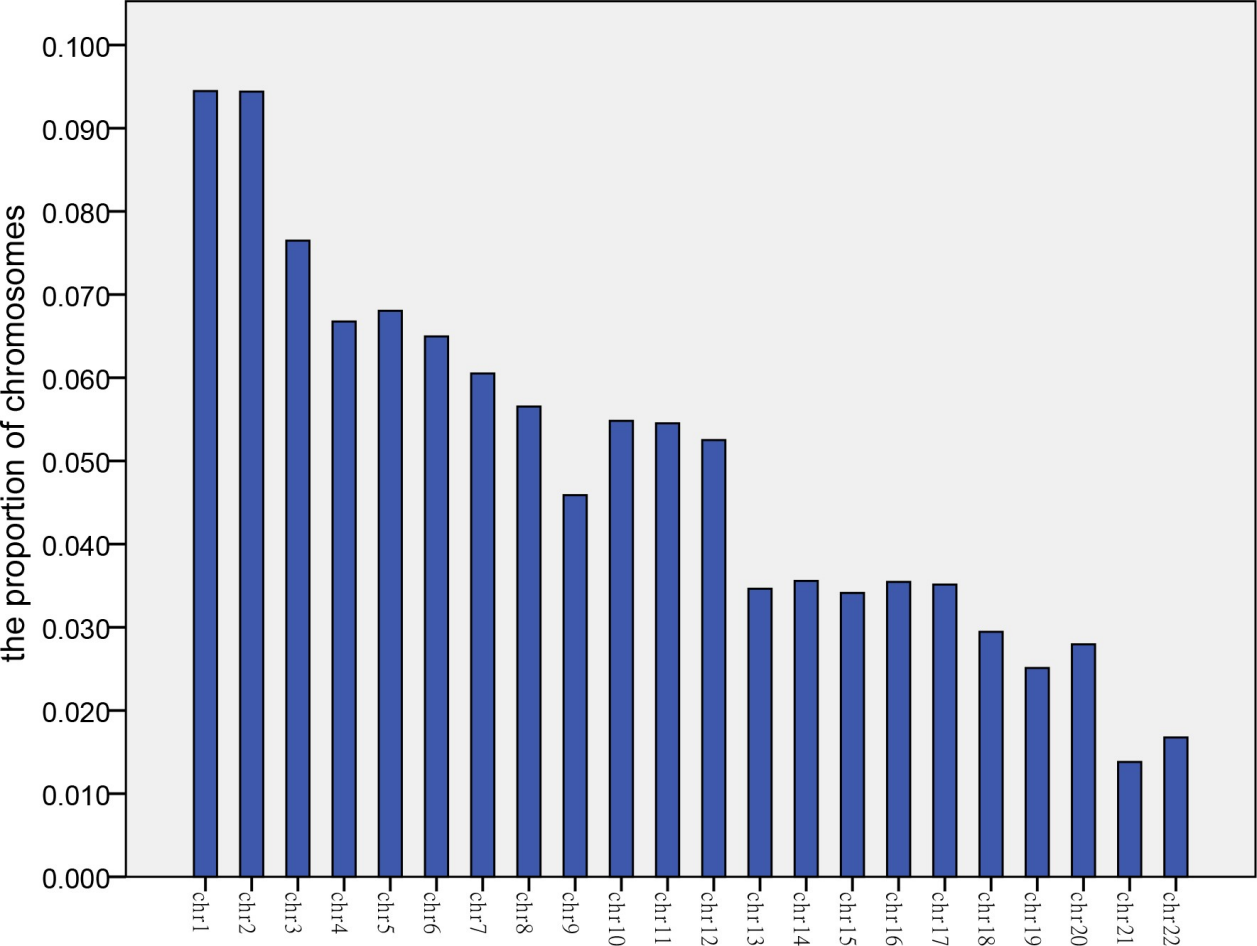
The proportion of autosomes. This bar chart shows the average proportion of autosomes in maternal plasma for 60 euploid samples.

**Fig 2:**
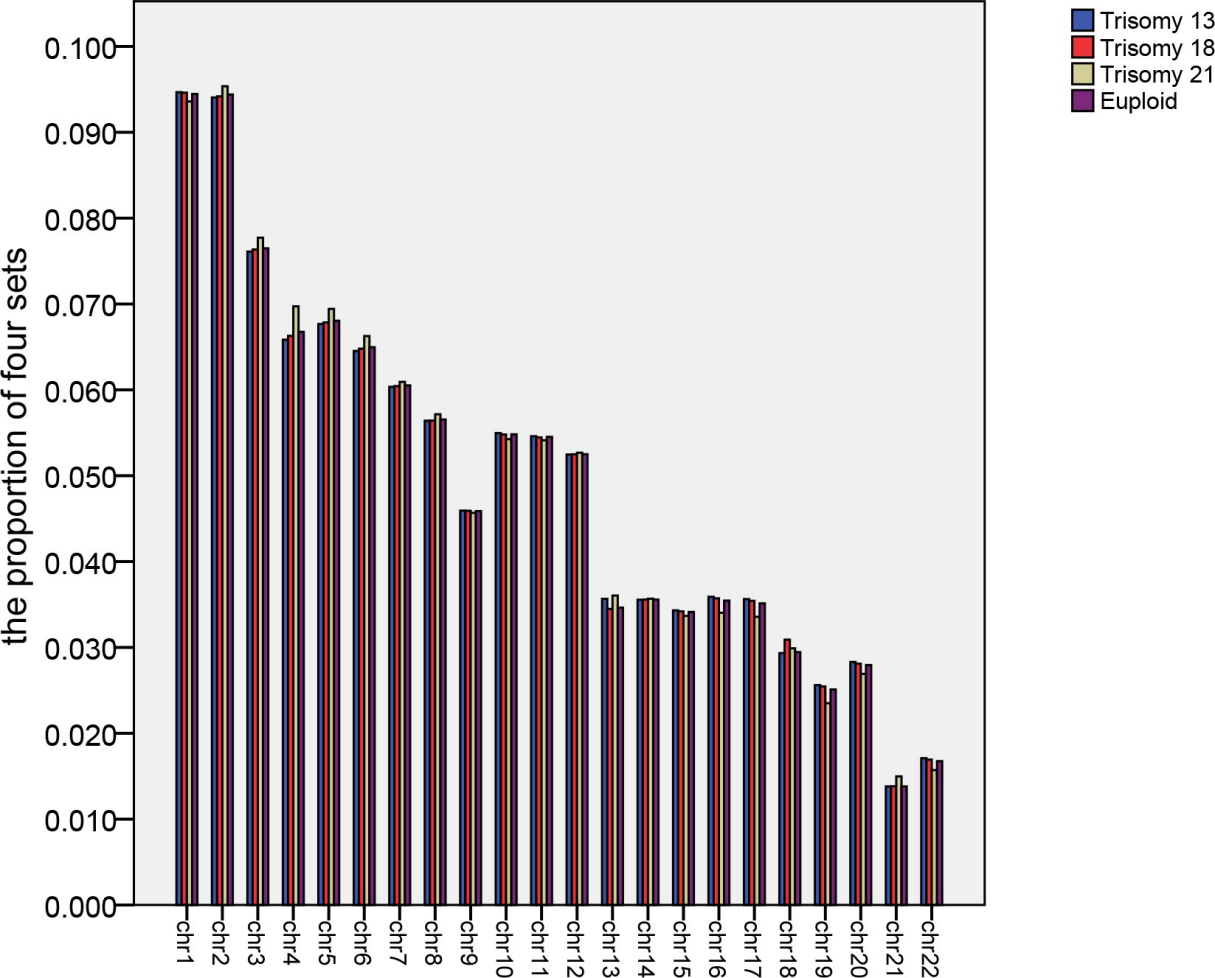
Comparison of four sets of data. Blue, red, yellow, and purple strips indicate trisomy 13, trisomy 18, Down syndrome, and euploid pregnancies, respectively.

Next, we calculated the average value of each chromosome for 60 euploid samples originating from maternal plasma DNA (Fig 1). Similarly, we calculated the average value of each chromosome for 21 cases with a trisomy 13 fetus, 27 cases with a trisomy 18 fetus, and 36 cases with a trisomy 21 fetus. We compared the proportions of these four records represented them graphically (Fig 2).

Fig 1 indicates that the proportion of DNA fragments from each corresponding autosome ranged from about 2% to 9%. We found out that the proportion of 9 chromosomes from chromosome 7 to chromosome 15 was almost the same, except for chromosome 13 in Fig 2. The three ratios corresponding to chromosomes 13, 18 and 21 were of particular interest. Among the four groups of values corresponding to chromosome 13, the average of samples with a 13 trisomy and a 21 trisomy was higher. Among the four groups of values corresponding to chromosome 18, the average of 27 cases with a trisomy 18 fetus was highest. The average of 36 cases with a trisomy 21 fetus had a larger value than others among the four groups of values corresponding to chromosome 21.

We computed the average of 144 samples for each chromosome (all autosomes except chromosomes 13, 18, 21). We were not certain of the sex of all samples from maternal plasma DNA. The number of Y chromosome fragments in male fetuses was generally larger than that in female fetuses. If we determined the average value directly, it would lead to a large error in the calculation result. The sex chromosome calculation data should thus be discarded. The method we used could also detect the sex of the fetus in this study. We noted the average value and standard deviation of each chromosome in S2 Appendix.

### Exploring the most suitable range

The primary research question of the study was to explore the most suitable range of cell-free DNA fragment lengths for detecting the three fetal chromosomal aneuploidies with a high accuracy.

We gradually narrowed the range of the interval via a traversing algorithm, and finally settled on a range of DNA fragment lengths of 80 to 155 bp (details of sample collection and processing available in supplementary files). We first calculated the value of the lower boundary from 30 to 130 in 10 bp steps and the value of the upper boundary from 150 to 170 in 5 bp steps. We obtained the most suitable range in the 114 sample cases through the calculated results. The lower limit range started from 75 to 85 bp and the upper limit range started from 150 to 155 bp; the resulting accuracy was relatively high throughout the range.

**Fig 3:**
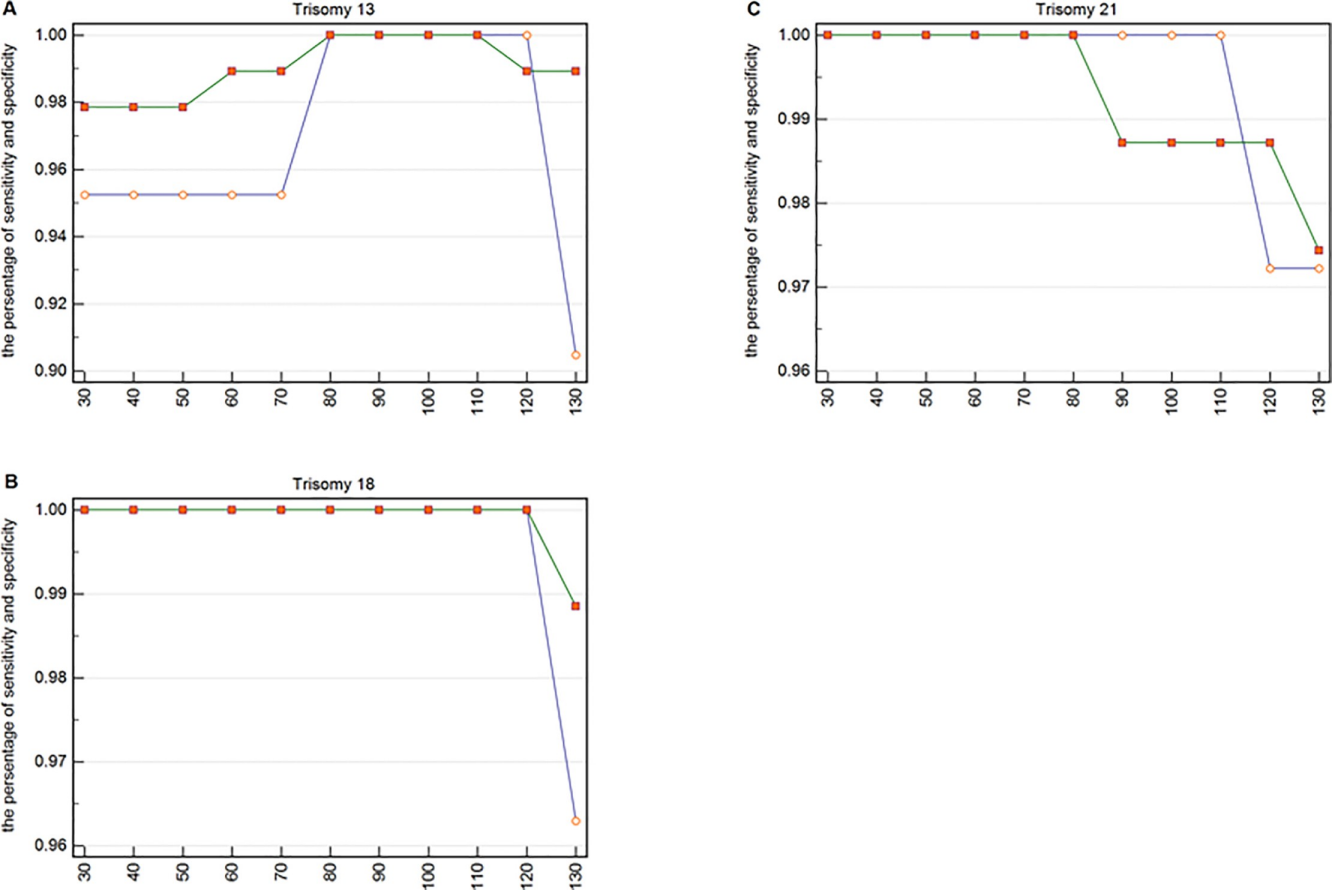
The specificity and sensitivity of each lower boundary for trisomy 13, 18, and 21. (**A**) The specificity and sensitivity for 21 pregnancies with trisomy 13 and for 93 pregnancies without trisomy 13. The small circles represent sensitivity and the small squares represent specificity. (**B**) The specificity and sensitivity for 27 pregnancies with trisomy 18 and for 87 pregnancies without trisomy 18. The small circles represent sensitivity and the small squares represent specificity. (**C**) The specificity and sensitivity for 36 pregnancies with trisomy 21 and for 78 pregnancies without trisomy 21. The small circles represent sensitivity and the small squares represent specificity.

We could infer the lower boundary by analyzing the specificity and sensitivity of these three chromosomal diseases shown in Fig 3. The results of the identification of Edwards syndrome using cell-free DNA fragments ranging from 30 to 120 bp had little change.

In Fig 3, the abscissa represents the value of the lower bound of each interval, and the upper bound was 150 bp. The two points of ordinate represents the sensitivity and specificity, respectively. For instance, the two points corresponding to the abscissa 30 represent the value of the sensitivity and specificity, respectively, when the range is 30 to 150 bp. We calculated the value of the lower boundary from 30 to 130 in 10 bp steps and observed the best intervals to be 80 to 110 bp for Trisomy 13, 30 to 120 bp for Trisomy 18, and 30 to 80 bp for Trisomy 21. The most suitable range we obtained was 80 to 150 bp, through the “AND” algorithm, and this was obtained with 150 bp as the upper bound.

**Fig 4:**
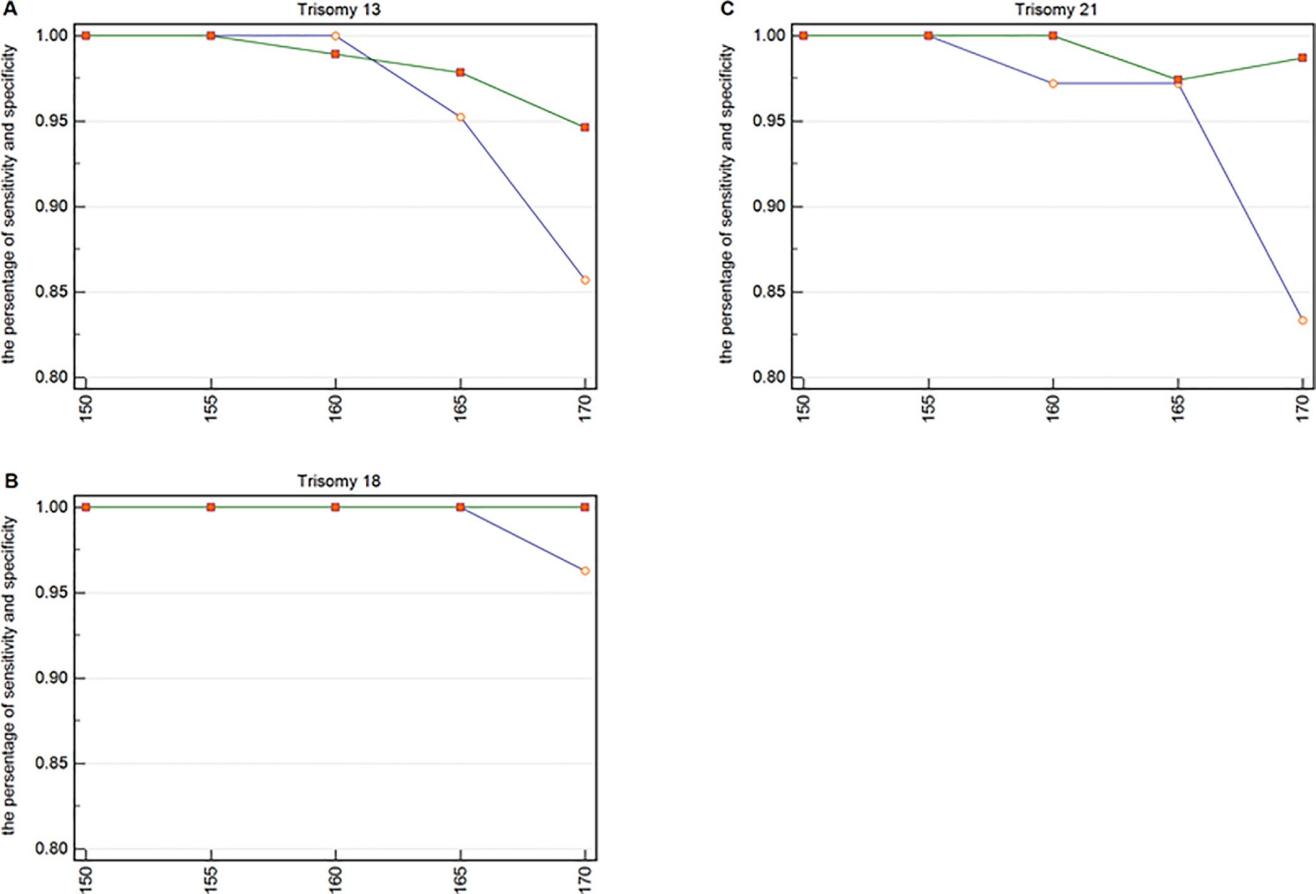
The specificity and sensitivity of each upper boundary for trisomy 13, 18, and 21. (**A**) The specificity and sensitivity for 21 pregnancies with trisomy 13 and for 93 pregnancies without trisomy 13. The small circles represent sensitivity and the small squares represent specificity. (**B**) The specificity and sensitivity for 27 pregnancies with trisomy 18 and for 87 pregnancies without trisomy 18. (**C**) The specificity and sensitivity for 36 pregnancies with trisomy 21 and for 78 pregnancies without trisomy 21.

Similarly, we could obtain the upper boundary by analyzing the specificity and sensitivity of these three chromosomal diseases shown in Fig 4.

In Fig 4, the abscissa represents the value of the upper bound of each interval, and the lower bound is 80 bp. The two points of ordinate represent the sensitivity and specificity, respectively. For example, the two points corresponding to the abscissa 150 represent the value of the sensitivity and specificity, respectively, when the range is 80 to 150 bp. We calculated the value of the upper boundary from 150 to 170 in 5 bp increments and observed the best interval to be 150 to 155 bp for Trisomy 13, 150 to 165 bp for Trisomy 18, and 150 to 155 bp for Trisomy 21. The most suitable range we obtained was 80 to 155 bp through the “AND” algorithm, and this was obtained using 80 bp as the lower bound.

From this, we obtained the values of the upper and lower bounds when determining the three most common autosomal aneuploidies. The accuracy and sensitivity of the DNA fragments between 80 bp and 155 bp was relatively high. Through the above calculations, the suitable range of DNA fragments length was found to be 80 to 155 bp for the sample set we used.

### Detection of fetal trisomies 18 and 21 based on the total range of fragment lengths

We randomly selected 30 sample cases with a euploid fetus originating from the sample set as training samples. We calculated a z-score for the target chromosome 18 and 21 for test samples using the following equation:

$$z-score_{chrN}=\frac{P_{chrN\_test}-meanP_{chrN\_training}}{SD_{chrN\_training}}$$

Where *P*_*chrN*_ is the proportion of the corresponding target chromosome; the mean *P*_*chrN*_*training*_ is the average value of 30 training samples; the *SD*_*chrN*_*training*_ is the standard deviation of 30 training samples.

**Fig 5:**
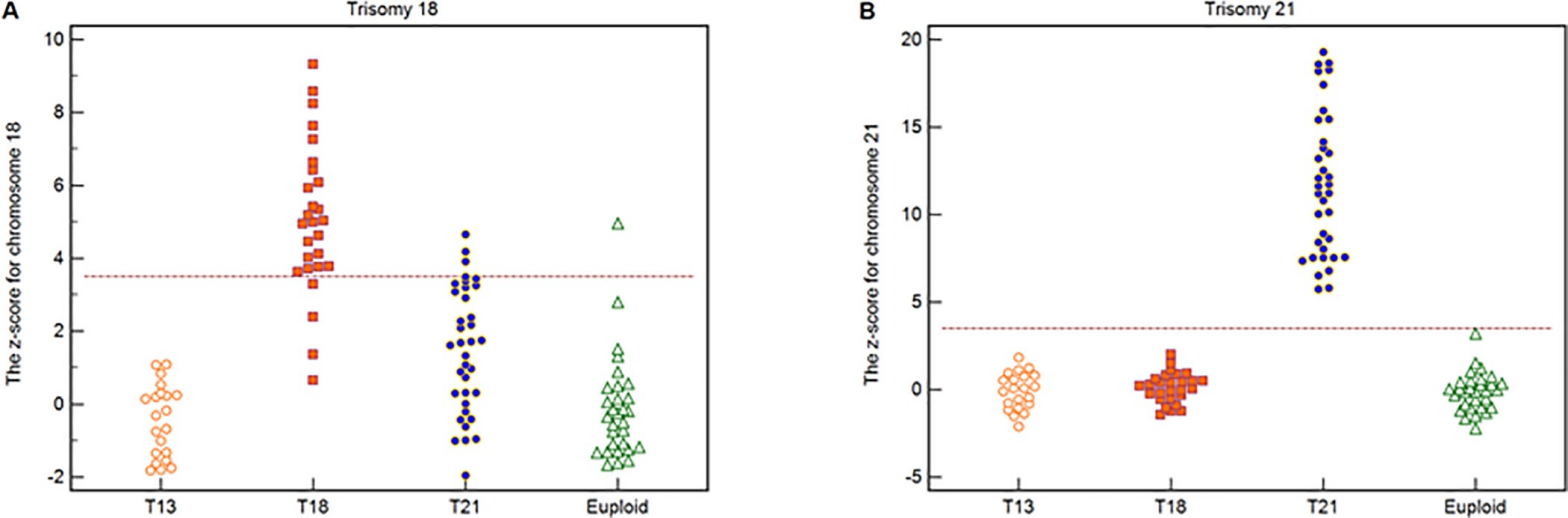
The z-scores for chromosomes 18 and 21. (**A**) The z-score of chromosome 18 for 114 test samples which included 21 trisomy 13, 27 trisomy 18, 36 trisomy 21, and 30 euploid cases. Dotted line denotes the z-score cutoff value of 3.5. (**B**) The z-score of chromosome 21 for 114 test samples which included 21 trisomy 13, 27 trisomy 18, 36 trisomy 21, and 30 euploid cases. Dotted line denotes the z-score cutoff value of 3.5.

Using a z-score cutoff value of >3.5, all 36 cases each with a trisomy 21 fetus could be correctly distinguished and 78 non-trisomy 21 cases could be correctly identified (Fig 5). The sensitivity and specificity both were 100%. Twenty-three out of 27 cases each with a trisomy 18 fetus could be correctly distinguished, and 83 out of 87 non-trisomy 18 cases could be correctly identified (Fig 5). The sensitivity was 85.2% and the specificity was 95.4%.

### Detection of fetal trisomies 13, 18 and 21 by the certain range of fragment lengths

In the previous method, the accuracy we obtained for the total range of fragment lengths was not very high. However, the specificity and sensitivity of the DNA fragments between 80 and 155 bp was relatively high. We would use the range, which was obtained through previous calculation to detect the three most common autosomal aneuploidies. If a fetus suffers from an autosomal aneuploidy, it would have one chromosome more than a euploid fetus. The number of fetal DNA fragments from the extra chromosome would thus increase.

We used the ratio to represent the relationship between the target chromosome (chromosomes 13, 18 and 21) and the other chromosomes (all autosomes except chromosomes 13, 18 and 21), denoted by *R*_*chrN*_, using the following equation:

$$R_{chrN}=\frac{P{\left(80-155\right)}_{chrN}}{P{\left(80-155\right)}_{chrother}}$$

Where *P*(80−155)_*chrN*_ denotes the proportion of DNA fragments originating from the goal chromosome with sizes ranging from 80 bp to 155 bp and *P*(80−155)_*chrother*_ denotes the proportion of DNA fragments originating from the other chromosomes with sizes range from 80 bp to 155 bp.

Then, we randomly selected 30 sample cases with a euploid fetus originating from the sample set as training samples. We used an improved z-score to distinguish the fetus sample cases, defining a new formula as follows:

$$z-score_{chrN}=\frac{R_{chrN\_Test}-meanR_{chrN\_Training}}{SD_{}\;R_{chrN\_Training}}$$

Where *R*_*chrN*_*Test*_ is the *R*_*chrN*_ for the test sample, *meanR*_*chrN*_*Training*_ is the mean *R*_*chrN*_ of the training samples, and *SDR*_*chrN*_*Training*_ is the SD of the *R*_*chrN*_ of the training samples.

**Fig 6:**
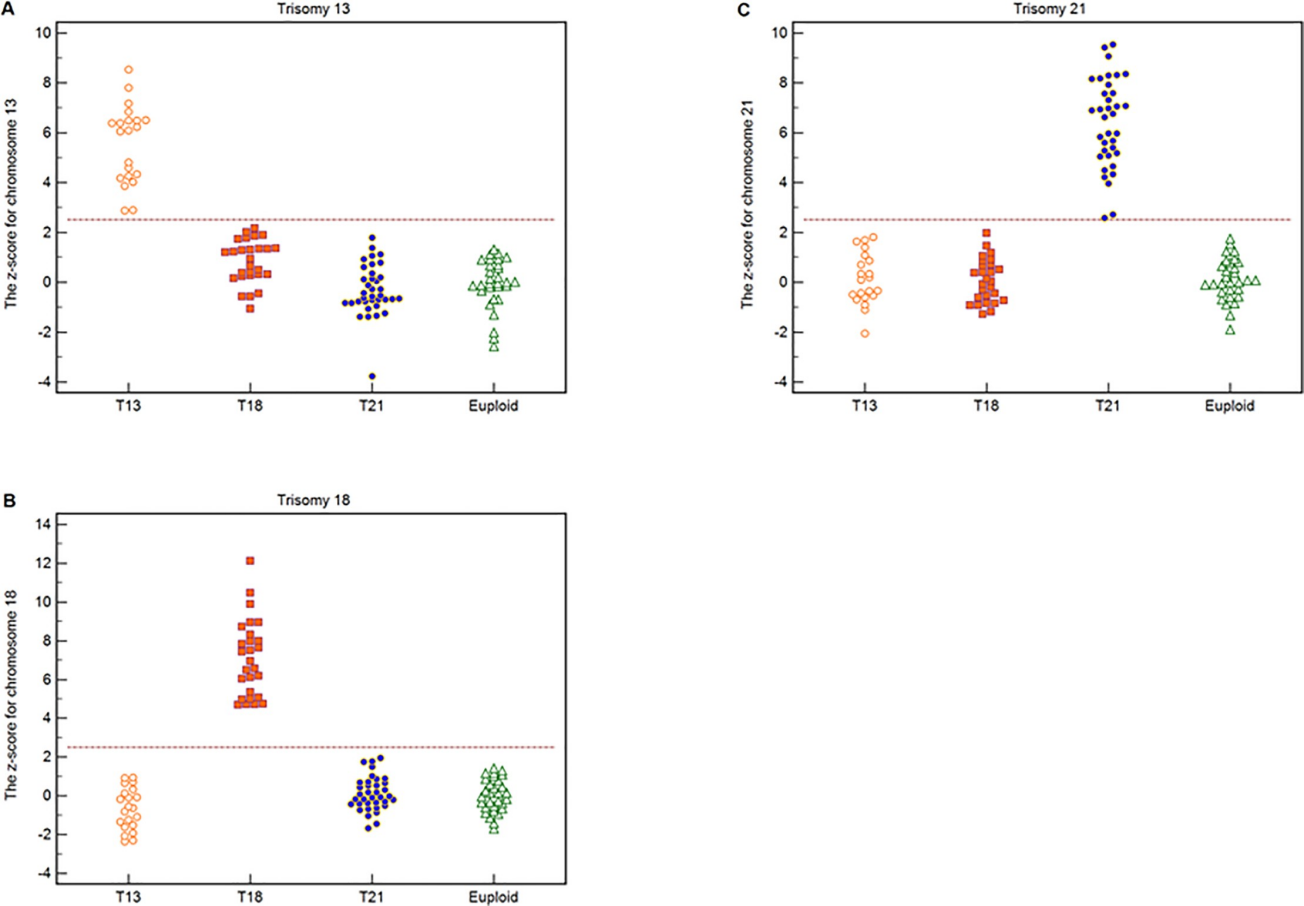
The z-scores for chromosomes 13, 18 and 21. (**A**) The z-score of chromosome 13 for 114 test samples, which include 21 trisomy 13, 27 trisomy 18, 36 trisomy 21, and 30 euploid cases. Dotted line denotes the z-score cutoff value of 2.5. (**B**) The z-score of chromosome 18 for 114 test samples, which include 21 trisomy 13, 27 trisomy 18, 36 trisomy 21, and 30 euploid cases. (**C**) The z-score of chromosome 21 for 114 test samples, which include 21 trisomy 13, 27 trisomy 18, 36 trisomy 21, and 30 euploid cases.

Using a z-score cutoff value of >2.5, all 21 cases each with a trisomy 13 fetus could be correctly distinguished and 93 non-trisomy 13 cases could be correctly identified (Fig 6A). The sensitivity and specificity both were 100%. All 27 cases each with a trisomy 18 fetus could be correctly distinguished and 87 non-trisomy 18 cases could be correctly identified (Fig 6B). There was an outlier replaced by the average value. The sensitivity and specificity were both 100%. All 36 cases each with a trisomy 21 fetus could be correctly distinguished and 78 non-trisomy 21 cases could be correctly identified (Fig 6C). The sensitivity and specificity both were 100%.

## Discussion

In the study, we calculated the proportions of all 24 chromosomes by observation and comparison. In general, the ratio of each chromosome from sequencing samples tends to be consistent. When a fetus had autosome aneuploidy, its corresponding chromosome ratio would increase. We know that if there are extra or missing chromosomes, the number of corresponding fetal DNA fragments will increase or decrease [24–26]. For instance, the proportion of chromosome 21 in a sample from a pregnant woman carrying a trisomy 21 fetus is higher than in a normal maternal plasma sample [27]. We can use this principle to predict multiple chromosome diseases. This method we used is simple and effective, and can be used as a first-tier screening test to determine multiple types of fetal autosomal aneuploidies.

We explored the values of the upper and lower bounds when determining the three most common autosomal aneuploidies and finally settled on a size range from 80 bp to 155 bp. We suspected that cell-free fetal DNA fragments are mainly concentrated in this size range through this processing. With the accumulation of data for all 24 human chromosomes, all genetic problems are expected to become detectable, allowing for accurate screening during pregnancy to ultimately reduce the incidence of fetal birth defects [28–30].

Noninvasive prenatal testing is now widely used in the medical field. The basic principle of NIPT is the need to extract cell-free DNA from the plasma of pregnant women to perform high-throughput sequencing [31,32]. Combined with economic and health data, NIPT can be used as a sequential screening program for traditional detection techniques. At present, the clinical application of NIPT can generally make a clear analysis of the three most common autosomal aneuploidies [31–33].

Our analysis showed that this method improves the accuracy of detecting the three most common autosomal aneuploidies to some extent. Our method requires a full range of genome sequencing, and massively parallel sequencing is currently expensive. The applicability of the method that is used as a first tier screening test requires a formal investigation of its diagnostic performance and cost-effectiveness. Furthermore, the amount of cell-free fetal DNA in maternal plasma is extremely small and the length of the pregnancy may affect the accuracy of diagnosis. The method is based on the length of the fragment and the corresponding number. Therefore, it is ensured that the cell-free fetal DNA in the plasma of pregnant women reaches a certain concentration. It is worthy of recognition that the results of this study provide the support for existing clinical data. In order to make this method apply to clinical diagnosis, we will apply our approach to the latest clinical data for improve the feasibility and make it be put into clinical diagnosis as soon as possible.

## Conclusions

In conclusion, numerical experiments demonstrate that our method is effective and efficient. Research into the size range of cell-free fetal DNA fragments is important for the performance of NIPT and its clinical assessment. This study suggests that most of the cell-free fetal DNA fragments are between 80 and 155 bp in length, which may serve as a valuable reference point for future research. The results of sample collection and processing are supplemented S1 Files.

## Supporting information

S1 Appendix The proportion of chromosomes. The appendix is the proportion of every corresponding chromosome for 144 samples.
(XLSX)

S2 Appendix The mean and SD of chromosomes. The appendix is the average value and standard deviation of chromosomes for 144 samples.
(XLSX)

S1 Dataset Sample set. The sample set included 21 cases each with a trisomy 13 fetus, 27 cases each with a trisomy 18 fetus, 36 cases each with a trisomy 21 fetus, and 60 cases each with a euploid fetus.
(XLSX)

S1 Files Supplementary files. The supplementary files include the result of sample collection and processing.
(ZIP)
